# Contemporary divergence in early life history in grayling (*Thymallus thymallus*)

**DOI:** 10.1186/1471-2148-11-360

**Published:** 2011-12-13

**Authors:** Gaute Thomassen, Nicola J Barson, Thrond O Haugen, L Asbjørn Vøllestad

**Affiliations:** 1 Centre for Ecological and Evolutionary Synthesis, Department of Biology, University of Oslo, P. O. Box 1066 Blindern, NO-0316 Oslo, Norway; 2 Oppland County Governor, P.O. Box 987, NO-2626 Lillehammer, Norway; 3 Norwegian Institute for Water Research, Gaustadalléen 21, NO-0349 Oslo, Norway; 4 Norwegian University of Life Sciences, Department of Ecology and Natural Resource Management, P. O. Box 5003, NO-1432 Ås, Norway

## Abstract

**Background:**

Following colonization of new habitats and subsequent selection, adaptation to environmental conditions might be expected to be rapid. In a mountain lake in Norway, Lesjaskogsvatnet, more than 20 distinct spawning demes of grayling have been established since the lake was colonized, some 20-25 generations ago. The demes spawn in tributaries consistently exhibiting either colder or warmer temperature conditions during spawning in spring and subsequent early development during early summer. In order to explore the degree of temperature-related divergence in early development, a multi-temperature common-garden experiment was performed on embryos from four different demes experiencing different spring temperatures.

**Results:**

Early developmental characters were measured to test if individuals from the four demes respond differently to the treatment temperatures. There was clear evidence of among-deme differences (genotype - environment interactions) in larval growth and yolk-to-body-size conversion efficiency. Under the cold treatment regime, larval growth rates were highest for individuals belonging to cold streams. Individuals from warm streams had the highest yolk-consumption rate under cold conditions. As a consequence, yolk-to-body-mass conversion efficiency was highest for cold-deme individuals under cold conditions. As we observed response parallelism between individuals from demes belonging to similar thermal groups for these traits, some of the differentiation seems likely to result from local adaptation

**Conclusion:**

The observed differences in length at age during early larval development most likely have a genetic component, even though both directional and random processes are likely to have influenced evolutionary change in the demes under study.

## Background

Large changes in environmental conditions put populations at risk of becoming maladapted, thereby leading to negative population growth and potential extinction [1]. Such large changes in environmental conditions may be due to anthropogenic activity [2], but may also be experienced as individuals invade a novel environment. For ectotherms in general, temperature is considered to be by far the most important external factor, controlling much of the variation seen in embryonic ontogenetic rates [3-5]. Following an environmental perturbation a population may be maladapted to the new conditions. If populations cannot rapidly move towards the new evolutionary optimum they may risk extinction [6].

Modification of traits may happen through purely plastic processes or through evolutionary change [7-9]. Phenotypic plasticity is defined as the ability of a single genotype to develop different phenotypes under different environmental conditions [7]. Norms of reaction (i.e., the variation in trait value across environmental gradients) are commonly used to illustrate the phenotypic plasticity of genotypes (reviewed in [10]). When a perturbation to the environment is outside of the normal range of environmental fluctuations, it is expected that evolutionary change will be required to reach the new fitness optimum [6]. In many situations, for example where there is substantial spatial habitat heterogeneity, the norms of reaction may be the target of selection [11]. Divergence in the slope and/or elevation of reaction norms may develop among populations occupying habitats that differ in their environmental characteristics. Since many traits depend on conditions experienced earlier in life, the reaction norm concept has been extended to include developmental processes [12]. These developmental reaction norms may be the target of selection, where individuals that most optimally modify their developmental trajectories under varying environmental conditions are selected.

A change in the population reaction norm requires genetic change, either through stochastic processes like genetic drift, or through adaptive evolution. Today it is realized that ecological and evolutionary dynamics do not necessarily operate on vastly different timescales [13-15]. A mounting number of studies provide evidence of evolutionary change over contemporary time scales [16,17].

**Figure 1:**
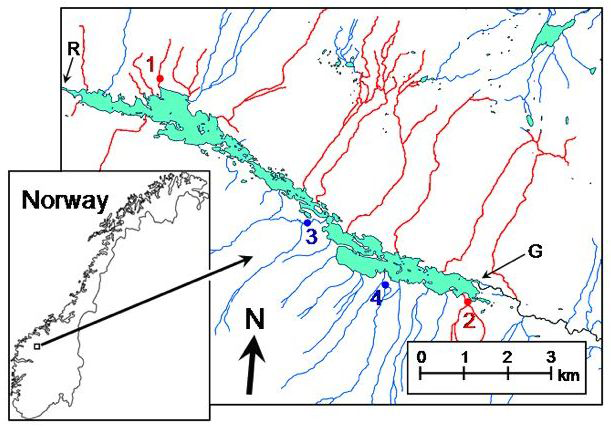
**Map of the Norwegian lake Lesjaskogsvatnet**. Tributaries colored blue are defined as large-and-cold (LC) tributaries while the tributaries colored red are defined as small-and-warm (SW) tributaries. 1 = Steinbekken (SW), 2 = Sandbekken (SW), 3 = Valåe (LC), 4 = Hyrjon (LC), R = Rauma outlet and G = Gudbrandsdalslågen outlet. For further details see text.

In salmonid fishes, thermal adaptation is thought to be important, especially during the egg-stage and subsequent phases following hatching. For fish larvae in general, size appears to be positively correlated with early-life survival [18-20]. This rule has been termed the "bigger-is-better"-hypothesis; for fish larvae, performance is size-dependent to a stronger degree than it is age-dependent, or dependent on any other measure of biological time [21,22]. The size effect is particularly strongly expressed under conditions of high competition, since there tends to be strong dominance hierarchies in juvenile salmonids [23,24]. Strong directional, size-dependent selection should thus lead to increased individual development rates in a population. We here investigate if that is the case in several demes of grayling *Thymallus thymallus *in an alpine Norwegian lake (Figure 1).

Grayling, a spring spawning salmonid fish, colonized the lake Lesjaskogsvatnet through a man-made connection in the upper reaches of the river Gudbrandsdalslågen that was opened in the late 1880s (20-25 generations ago). Subsequent closing of the connection made further immigration of grayling difficult [25]. Genetic evidence suggests that Lesjaskogsvatnet was colonized by a low number of individuals from Gudbrandsdalslågen [26]. Since the colonization event, the grayling have established more than 20 demes spawning in separate tributaries to the lake [27]. It is likely that grayling return to their natal tributary to spawn. Lacustrine populations of grayling prefer to move to running water to spawn and are known to return to their natal stream with high fidelity [28].

During spring, the south-facing slopes of Lesjaskogsvatnet receive considerably more sunlight and are less steep than the shaded north-facing slope resulting in more rapid spring warming. In addition, small tributaries tend to warm more rapidly than large ones. As a result of these differences in aspect coupled with size differences among tributaries, tributaries can generally be classified as either large-and-cold (LC) or small-and-warm (SW) [27]. Generally, spring ice-melt commences earlier in SW tributaries than in LC tributaries, and SW tributaries have a higher temperature than LC tributaries through spring and early summer [29]. Typically, mean daily June- July water temperatures differ by 1-1.5 °C among LC and SW streams, which add up to substantial differences in accumulated temperature sums over the stream phase.

Haugen and Vøllestad [30] conducted a common-garden study of several early growth and developmental traits of grayling in the same area, but on an inter-lake scale. They investigated differences between the Lesjaskogsvatnet population and two other populations recently derived from Lesjaskogsvatnet grayling. In their study, they discovered apparent adaptations in many of the traits. Based on the same data, evidence for divergence in time to hatching was found between two of the populations [31]. These results demonstrate that in the absence of gene flow local adaptation may progress significantly over less than a 20-generation time span. Here, we use a common environment approach at three different temperature conditions to investigate how temperature reaction norms for early life history traits vary for grayling from two cold and two warm streams where gene flow among streams is possible.

In high latitude mountain lakes like Lesjaskogsvatnet the growth season is short. For north-temperate fishes the first winter is usually a period of energy deficit [32], potentially selecting for faster growth. The selection intensity is expected to be strongest for the demes with the shortest growth season (i.e. LC demes). Individuals from these demes are expected to compensate for the shorter growth season with a higher *general *capacity for growth [33].

As grayling occupy divergent habitats during development it is possible that evolutionary divergence could result. However, one should bear in mind that this divergence is observed under conditions of continuing gene flow and potential meta-population dynamics [34,35]. The grayling could have responded to the differing environmental conditions by evolutionary changes; alternatively, the grayling could have a plastic response common to individuals of all demes [9]. If the latter is the case, reaction norms for all demes should appear more or less identical. If reaction norms differ this would be indicative of genetic effects.

In this study, we test if there is evidence for evolution in early life-history traits in larval grayling from four different spawning demes that are experiencing different environmental selection pressures. To contrast the development of larval grayling from different demes a common-environment experiment was conducted. In the experiment, individuals from four tributaries (two LC, two SW) were subjected to three experimental temperatures and studied from fertilization to swim up, the stage when the juveniles emerge from the gravel and become free-living [36].

## Results

### Initial egg size

Mean initial egg size (measured as egg diameter, mm) differed significantly among demes (one-way ANOVA: F_3, 116 _= 7.13, P = 0.0002, R^2^= 0.123). The largest and the smallest eggs were found in the warm stream demes (mean ± 2se; Hyrjon: 3.88 ± 0.06; Valåe: 3.94 ± 0.07; Steinbekken: 4.03 ± 0.09; Sandbekken: 3.79 ± 0.09).

### Larval length through time

**Figure 2:**
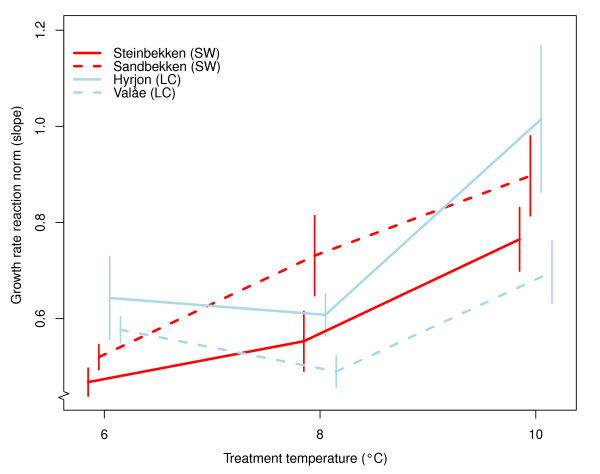
**Temperature reaction norms**. Reaction norms illustrating the effect of treatment temperature on growth rate (estimated as the slope ± se of deme-wise linear regressions of ln(length) on degree-days for each temperature) for the four demes studied. Bold lines indicate SW demes.

Variation in larval length from hatching to swim-up clearly differed among demes and treatments, and there was a significant three-way interaction among treatment, deme and degree-days (Table 1, Additional file 1; details on results from each deme-treatment combination are presented in Additional file 2). All model terms, except for the treatment*deme interaction, significantly influenced larval length. For the two SW demes growth rate (estimated as the slope of linear regression of length (ln-transformed) and time measured as degree-days) increased with increasing treatment temperature (visualized in Figure 2). For the LC demes, growth rates tended to be lower at the medium treatment temperature. At the coldest treatment temperature (5.8°C), growth rates for LC deme individuals (Hyrjon: 0.643 ± 0.086; Valåe: 0.577 ± 0.027) were higher than those for SW deme individuals (Sandbekken: 0.520 ± 0.026; Steinbekken: 0.468 ± 0.029, Figure 2); the non-overlapping standard errors between cold and warm demes suggest significant differences in growth rate at this temperature. At the remaining temperatures there was no apparent deme-type related pattern in the rank order of growth rates. Grayling from all demes attained their highest growth rate in the warmest treatment temperature (10.0°C).

**Table 1:** Effect test for variation in larval length over time.

Model term	Df	Sum of squares	F-ratio	P-value
ln°d	1	2.571	1190.14	<0.001
Deme	3	0.160	24.62	<0.001
Treatment	2	0.982	227.27	<0.001
Deme*ln°d	3	0.034	5.21	0.002
Treatment*Deme	6	0.028	2.15	0.122
ln°d*Treatment	2	0.086	19.95	<0.001
Treatment*Deme*ln°d	6	0.031	2.42	0.026
Replicate [Deme, Treatment]	12	0.004		

Summary test statistics for a linear mixed model with ln(larval length) as response variable and replicate as a random effect. Adjusted R^2^: 0.712. N = 709. The parameter estimates are available in table A1 in Additional file 1. Square brackets indicate the nesting structure. Layout details: °d is the degree days-covariate, an asterisk between model term indicates interaction effect, and Df indicates degrees of freedom for the relevant model term.

### Yolk sac size, consumption and conversion efficiency

The rate of change in yolk sac size over time clearly differed among demes and treatments (Table 2, Additional file 1; details on results from each deme-treatment combination are presented in Additional file 3). The significant two-way interactions among treatment, deme and degree-days indicated that the reaction norms differed significantly. For LC deme grayling, yolk consumption rate appeared to be highest in the intermediate treatment temperature (8.1°). The highest energy consumption rate for SW deme individuals was found at the lowest treatment temperature. The fully factorial model included quadratic ln-transformed degree-day terms as initial models indicated that the consumption rate decreased with increasing degree-days.

**Table 2:** Effect test for variation in larval yolk sac size over time.

Model term	Df	Sum of sq	F-ratio	P-value
Treatment	1	8.33	28.96	<0.0001
Deme	3	10.25	23.77	0.0022
(ln°d)^2^	2	381.10	1325.30	<0.0001
Treatment*Deme	3	4.31	4.99	0.0001
Treatment*(ln°d)^2^	6	10.96	19.06	<0.0001
Deme*(ln°d)^2^	2	8.66	10.04	<0.0001
Treatment*Deme*(ln°d)^2^	6	0.90	0.52	0.8997
Replicate [Deme, Treatment]	12	1.929		

Summary test statistics for a linear mixed model with ln(yolk sac area) as response variable. Replicate is random effect. Adjusted R^2 ^= 0.713, N = 713. The parameter estimates are available in table A1 in Additional file 1. Square brackets indicate the nesting structure. Layout details: The (ln°d)^2 ^notation indicates the total polynomial effect (i.e., ln°d + (ln°d)^2^) was fitted as a orthogonal polynome, an asterisk between model terms indicates interaction effect, and Df indicates degrees of freedom for the relevant model term.

There was little support for thermal group parallelism in the yolk consumption pattern as fitting the fully factorial model with the deme effect substituted by group increased AIC by 74 units (full model with deme effect; AIC = 670, full model with temperature group effect; AIC = 744).

**Figure 3:**
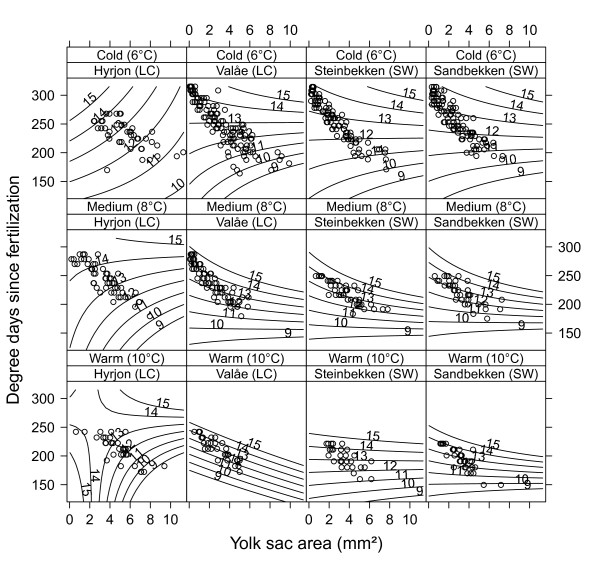
**Yolk conversion efficiency**. Estimated deme-wise body length (isoclines, value = mm) as function of yolk-sac area and degree-days since fertilization at different treatment temperatures. The estimates are retrieved from a linear mixed effects model where replicate variation was modeled as a random effect, nested under deme. Dots represent measured yolk sac values at given degree-days.

The yolk-to-body-size conversion analysis, using linear mixed effects (LME) models (Figure 3, Table 3), showed that the two LC demes were most efficient in transferring yolk to body size under the cold and medium treatment regimes, whereas there was little differentiation in efficiencies under the warm regime. Using this rather complex model, we can predict larval length at a given yolk sac area (here 1 mm^2^) under the various experimental conditions. For instance, assuming an almost reabsorbed yolk sac with an area of 1 mm^2^, a cold treatment and at 270 degree-days, the estimated larvae lengths would be 14.9, 13.1, 12.7, and 12.6 mm for Hyrjon, Valåe, Steinbekken and Sandbekken, respectively. Under the warm treatment, still assuming a yolk-sac area of 1 mm^2^, but now under the more warm-regime relevant 230 degree-days, larval lengths were estimated to be 14.2, 13.8, 14.6, and 14.2 mm. The general variance inflation factor (GVIF; see Methods) for the interaction effect between yolk-sac area and degree-days (GVIF_°d*ys area _= 9.52) was slightly lower than the recommended cut-off value of 10 [37]. However, there was evidence of non-trivial collinearity for this interaction effect within temperature regimes (GVIF_°d*ys area*treatment*deme _= 28.9). When substituting the deme effect with a thermal group effect, the AIC decreased by 113 units, indicating that demes belonging to the same thermal group displayed parallelism in their temperature reaction norms for yolk-to-body-size conversion pattern (full model with deme effect; AIC = 1536, full model with temperature group effect; AIC = 1423).

**Table 3:** Effect test for variation in conversion efficiency.

Model term	Df	Sum of sq	F-ratio	P-value
YS-area	1	471.64	1575.42	<0.0001
°d	1	85.09	284.22	<0.0001
Deme	3	3.54	3.95	<0.0001
Treatment	2	29.01	48.45	<0.0001
YS-area *°d	1	31.39	104.86	<0.0001
YS-area *Deme	3	11.80	13.13	<0.0001
°d*Deme	3	7.54	8.39	0.0056
YS-area *Treatment	2	6.50	10.86	<0.0001
°d*Treatment	2	6.12	10.22	0.0010
Deme*Treatment	6	5.32	2.96	0.0077
YS-area *°d*Deme	3	0.76	0.85	0.4679
YS-area *°d*Treatment	2	2.64	4.41	0.0125
YS-area *Deme*Treatment	6	1.67	0.93	0.4746
°d*Deme*Treatment	6	5.08	2.83	0.0101
YS-area *°d*Deme*Treatment	6	2.25	1.25	0.2772
Replicate [Deme, Treatment]	12	0.05		

Summary test statistics for a linear mixed model with ln(length) as response variable. In order to test for yolk-to-body-size conversion efficiency yolk-sac area (YS-area) is included as predictor variable. Replicate, nested under treatment and deme, is random effect. Adjusted R^2^: 0.744. N = 707. Square brackets indicate the nesting structure. Layout details: °d is the degree days-covariate, an asterisk between model term indicates interaction effect, and Df indicates degrees of freedom for the relevant model term.

## Discussion

The results presented in this study demonstrate genetic differences in growth-related traits between grayling from four demes within Lesjaskogsvatnet. Grayling from different demes responded differently to changes in developmental temperature, with respect to growth in length and yolk-to-body-size transition efficiency. This appeared as differentiated temperature-related reaction norms for these traits. A major part of the among-deme variation in growth-related traits is likely to be genetically based since the results were obtained from a common-environment experiment. Parallelism of developmental phenotypes between demes belonging to similar thermal groups indicates that the differentiation is consistent with local adaptation.

We found significant among-deme variation in growth, estimated as the change in length over time, and in how yolk was allocated to growth. As expected from previous work ([38] and references therein), these traits were also significantly influenced by temperature. However, the different demes responded differently to the treatment temperatures. Haugen and Vøllestad [30] also discovered similar differences when testing three grayling populations having the same ancestry, but experiencing vastly different environmental conditions.

The differences in the growth rate estimates for LC deme individuals relative to the SW individuals may, to some extent, be interpreted as indicative of local adaptation. Larvae from the LC demes grow faster than SW deme individuals under experimental conditions comparable to what is observed in the LC deme habitats (i.e., the cold treatment temperature, Figure 2). LC deme individuals also have more efficient yolk-to-body size transition efficiencies under cold and medium temperature conditions (Figure 3). All grayling demes originate from fish spawning downstream in the river Gudbrandsdalslågen. Overall, temperatures in Gudbrandsdalslågen is comparable to the temperatures in the SW streams, indicating that only the LC deme individuals have been exposed to temperatures leading to strong selection. The full picture emerging from the data, however, does not fully correspond with predictions for the "bigger-is-better"-hypothesis or counter-gradient variation hypothesis [39,40]: at the medium and warm treatment temperatures, the rank order of demes appears not to follow a pattern that can be ascribed to conditions in their natal tributaries. Overall, there is strong evidence for genetically based differences in growth rate among demes. However, since the optimal strategy is unknown it is premature to decide whether the result is due to local adaptation or not. Further, there is a general trend in all demes for growth rates to increase with temperature, much in line with results from comparable studies [38].

Jensen *et al*. [41] found significant divergence in traits such as larvae length and growth rate of brown trout juveniles in a common-garden experiment, and suggested that this was due to local adaptation. However, as for the present study, fitness was not estimated so conclusions in this aspect are premature. In our study, differences in traits related to growth and energy utilization do not unambiguously correspond with predictions for local adaptation. Any lack of adaptation might be caused by evolutionary constraints, resulting from the history of the Lesjaskogsvatnet grayling. It is possible that the demes under investigation have not had equal opportunities for environmental adaptation and that equilibria have not been reached [34,35]. When investigating potential genetic effects in divergence and adaptation studies one should never overlook the potential importance of random genetic drift. Through the colonization process, the grayling of Lesjaskogsvatnet have gone through various severe bottlenecks [34,35]. Normally, bottlenecks are expected to reduce genetic variability and hence impede response to selection (e.g. [42,43]). However, because founder populations may contain a high fraction of the heterozygosity and additive genetic variance in quantitative characters that existed in a large source population, prospects for adaptation may not necessarily have to be impeded [44]. Potentially, genetic drift could complicate the interpretation of results since the outcome of evolution by genetic drift cannot be predicted (e.g. [45]). Further, in young systems such as in the Lesjaskogsvatnet grayling system gene flow and drift may vary in strength, leading to large among year variation in strength of selection [34]. Gene flow most often is considered to hinder local adaptation [46,47], however, this is not necessarily always the case [48,49]. Gene flow among inbred demes may counteract the effect of drift and increase the evolvability of these demes by introducing genetic variation, and as such enhance short-term adaptation [43]. However, in most situations where you have heterogeneous habitats and genotype-environment interaction effects, gene flow is predicted to constrain local adaptation [49,50].

The different traits studied here may be influenced by maternal effects. If such effects are present, and if they are physiological side effects and not consequences of adaptive evolution, they have the potential to confound our results. Potential maternal effects might be egg provisioning of energy and other important nutrients [51]. This cannot be directly accounted for in a study like ours, where the maternal environment is not controlled. One complicating factor is the time delay between maturation of gonads for LC and SW demes, potentially leading to quality differences in the eggs. In Haugen and Vøllestad's [30] study on grayling from the same area, egg size was found to have a significant contribution to the total female effect for three out of four early-life size traits and also on early survival rate. Our study design did not allow for family-level resolution. Egg size - a main determinant of maternal effects - did differ among the four demes. However, these differences cannot explain the parallelism in growth rates and energy use as both the largest and the smallest eggs were observed in the SW demes.

We have shown that several early life history traits have diverged among the four grayling demes studied here, and some of the divergence is consistent with adaptive predictions. Taken together with several related studies from the same area [29,30] this indicates that these traits can evolve rapidly and in spite of conditions that are generally assumed to constrain adaptation. We have shown that most of the demes studied have low genetic variability and have experienced bottlenecks and founder effects [34,35]. Gene flow among demes is strong and highly variable [34]. Additionally, some demes are small indicating that genetic drift may be strong. These conditions can potentially lead to strong genetic drift, a process that can constrain adaptive evolution [43,46,47]. A recent theoretical study does, however, show that dispersal, mutation and sexual reproduction may accelerate local adaptation in growing populations [49], especially when populations are small. However, these same processes may reduce local adaptation in the long run.

## Conclusions

In summary, we find evidence for significant among-deme variation in important early life-history traits in grayling from the Lesjaskogsvatnet. These differences have evolved during 20-25 generations in the face of gene flow, recent bottlenecks, and the fact that the grayling live in sympatry during most of their life. Some of these results are consistent with adaptation expectations as inferred from signs of parallelism, but we cannot rule out that other evolutionary or non-evolutionary processes have influenced the results.

## Methods

### Field sampling and experimental design

Adult spawning grayling were intercepted during their migration and captured using fyke nets. The traps were checked three times daily and captured fish were transferred to a holding pen upstream. Once capturing was complete, adult fish were anaesthetised with benzocaine and their weight and fork length were measured (see Table 4) before the fish were stripped of gametes. After recovery all fish were released upstream of the capture site. Gametes were transported on ice and under oxygen by car to the fish holding facility at the Veterinary Institute of Norway, Oslo (5 hours drive). Gametes were stripped from the SW stream populations on 12^th ^June 2007, and on 23^rd ^June the same year from the two LC stream populations.

**Table 4:** Information on parental fish.

Deme	Sex (N)	Weight (g)	Length (mm)
Steinbekken (SW)	♀ (20)	226.8 ± 130.3	287.6 ± 56.4
	♂ (16)	337.6 ± 89.3	333.25 ± 28.2
Sandbekken (SW)	♀ (17)	187.2 ± 85.6	268.6 ± 11.5
	♂ (11)	263.9 ± 100.7	305.6 ± 35.2
Hyrjon (LC)	♀ (4)	158.8 ± 90.4	254.8 ± 30.6
	♂ (3)	244.0 ± 61.2	299.3 ± 22.1
Valåe (LC)	♀ (20)	236.4 ± 106.8	284.4 ± 51.7
	♂ (24)	298.7 ± 82.2	327.0 ± 41.9

Descriptive data (number per tributary and sex; mean weight ± sd, mean length ± sd) for the mature grayling captured and stripped for gametes from the four different tributaries. SW are small and warm streams, whereas are LC are large and cold streams.

The eggs were pooled by deme using an equal volume (100 ml) of eggs from each female. The pooled batch of eggs from each deme was then split into a number of batches equal to the number of males from that deme. To avoid sperm competition and so maximise the number of families, each batch of pooled eggs was subsequently fertilized with sperm from one male. Following fertilization, the batches of eggs from each deme were mixed together again before they were partitioned into three treatment groups. Each treatment group was split into two replicates. Three separate experimental tanks containing water of three different temperatures (5.83 ± 0.43°C, 8.14 ± 0.29 °C, 10.02 ± 0.28 °C) were used. These temperatures were chosen to represent lower, medium and upper temperatures experienced by developing grayling larvae in nature [52], and thus we would be able to investigate norms of reaction covering the range of temperatures that grayling may be expected to tolerate. Mean summer (June - July) temperatures in the four streams investigated here differ strongly, with the two small and warm streams being approximately 1-1.5 ºC higher than the large and cold streams (Sandbekken 8.44 ± 0.52 (n = 5); Steinbekken 8.81 ± 0.60 (n = 4); Hyrjon 7.40 ± 0.94 (n = 8); Valåe 7.28 ± 0.69 (n = 0.69)). This adds to a large temperature-sum difference among streams during egg and larvae development.

Inflowing water was comprised of activated-charcoal filtered tap water. Temperatures were registered using temperature loggers (HOBO). Fertilized eggs were placed in porous containers suspended in the large treatment tanks as described in detail previously [30]. Each of the three tanks contained two replicate containers from each deme.

Animal sampling and experimentation were performed in compliance with the recommendations of National Animal Research Authority (permission ID 2008/7368.5) and under the supervision of authorized investigators.

### Data acquisition and analysis

Each day post fertilization approximately five individuals (eggs or larvae) were sampled from each deme in each treatment, alternating between the two replicates (Table 5). Sampling was done by randomly picking individuals out of the containers. The sampled eggs/larvae were carefully freed from water prior to fixation in buffered formalin in order to optimally retain physical characters [53]. Sampling was carried out every day over the 65 day experimental period with the exception of one day on which sampling was impossible due to technical problems.

**Table 5:** Sample sizes.

Deme	Replicate	Warm	Medium	Cold
Steinbekken (SW)	Rep 1	44 - 12	59 - 22	89 - 57
	Rep 2	34 - 16	51 - 20	82 - 48
Sandbekken (SW)	Rep 1	39 - 19	60 - 21	86 - 55
	Rep 2	35 - 16	52 - 18	81 - 51
Hyrjon (LC)	Rep 1	42 - 18	61 - 32	85 - 16
	Rep 2	36 - 15	52 - 24	62 - 29
Valåe (LC)	Rep 1	50 - 17	62 - 43	89 - 63
	Rep 2	40 - 16	60 - 27	80 - 56

**Sum**	Rep 1	175 - 66	242 - 118	349 - 191
	Rep 2	145 - 63	215 - 89	305 - 184

Number of eggs or larvae sampled during the common-garden experiment. Numbers given are total number of eggs and larvae sampled per deme, split by treatment (warm, medium, cold) and replicate (Rep 1, Rep 2) as well as totals pr treatment and replicate. SW are small and warm streams, whereas are LC are large and cold streams.

All samples were visually inspected to check for hatched individuals. Numbers of hatched individuals per sample was recorded. However, precise estimates of time at hatching could not be obtained because of large among-sample variation. Photographs of eggs and larvae were obtained using a Leica DC300 digital camera mounted on a Leica MZ8 stereo microscope connected to a computer and captured using IrfanView (version 3.99) http://www.irfanview.com/. One hundred and twenty eggs (5 eggs from each deme in each treatment for the first two days after fertilization) were photographed and initial egg size was estimated as the mean egg diameter measured in UTHSCSA Image Tool (version 3.0; http://ddsdx.uthscsa.edu/dig/itdesc.html). Out of the 2142 individuals sampled (see Table 5), 717 had hatched and these where measured for length and yolk sac area. All larvae were photographed twice; usually once with the left side facing the camera, and once with the right side facing the camera to allow repeated estimates of traits to be made. The mean of the two measurements was used in the analysis. Based on a random subsample of 100 individuals there was a mean deviation of 1.06% between the two length measurements done on each individual. The corresponding number for yolk sac area estimates was 7.77%. On certain occasions larvae were fixed in awkward positions and were too brittle to straighten out. If so, photographs in three positions (i.e. side facing up, back facing up and/or yolk sac facing up) were obtained in order to get the measurements needed. Length was measured from the tip of the cranium to the visible end of the notochord. ImageJ version 1.38 http://rsbweb.nih.gov/ij/download.html was used to obtain a measurement of the yolk sac area. Yolk sac area was estimated by fitting an ellipse to a polygon drawn along the edge of the yolk sac in order to estimate the longest and shortest possible axis of the fitted ellipse. Axis lengths obtained from the two photographs of each individual were averaged. The resulting estimates were used to estimate each individual's yolk sac area using the formula for the area of an ellipse (πab; a and b being one-half of the ellipse's major and minor axes, respectively).

Six individuals were completely excluded from the statistical analysis due to obvious disfigurement or severe damage during handling making any measurements uncertain. In four individuals the yolk sac was damaged or completely removed due to handling, making measurements only on length possible. One individual had an intact yolk sac, but a damaged body, making only measurement of the yolk sac area possible.

A one-way ANOVA on egg diameter with deme as effect was carried out, and a Tukey-Kramer HSD (honestly significant difference) test was used to compare deme means.

Continuous response variables were analyzed using linear mixed effect models (LME, [54]), fitted by means of the restricted maximum likelihood (REML) method. In the LME models fitted, the random effect of replicate was nested under deme and treatment. Both the response variables in question (larval length or yolk sac area) and the continuous covariate (degree-days (°d); a measure of time estimated as accumulated mean daily temperature) were logarithmically transformed to reduce heteroscedasticity. Individual linear regressions of length/yolk sac area on degree-days were fitted for each deme in every temperature (see Additional files 2 and 3). Full factorial models were used when analyzing the data (see equation 1).

(1) $$Y_{\text{ijk}}={ß}_{\text{1ij}}+{ß}_{\text{2}}d_{\text{ijk}}+{ß}_{\text{3ji}}*d_{\text{ijk}}+{\text{b}}_{\text{(ij)k}}+\varepsilon_{\text{ijk}}$$

Here, "*Y*" is the response variable, ßs correspond to fixed parameters under estimation and b_(ij)k _corresponds to the random effect of replicate (k) nested under treatment level i and deme j. Variables are provided in italics where "*°d*" indicates degree-days (ln-transformed), *ε*_ijk _represent the between individual variance (i.e., the residual variance) which is assumed to be normally distributed within replicates, and for a given link function. An asterisk indicates an interaction term. If non-linear trends were found in LME residual plots, new models including a squared term for the degree-days effect were fitted. In order to check for possible first-order temporal autocorrelation violations, Durbin-Watson tests were conducted for the fitted LME models.

In order to explore differentiation in the yolk-to-body-size conversion efficiency (see [29]), we fitted LME models where body length was predicted from yolk sac area and degree-days and with rearing temperature and deme as fixed effects. Replicates were nested under deme and temperature regime and were modelled as random effects. The full model structure was then compared with a comparable model where the deme structure was exchanged with a temperature-group (SW vs LC) structure, using Akaike Information Criterion (AIC)-values for comparison [55]. Due to collinearity between the predictor variables yolk-sac area and degree-days, information about variance inflation (assessed from the general variance inflation factor, GVIF) and the corresponding tolerance is presented together with the chosen model [56].

The statistical analyses of the data were carried out using the statistical software JMP 5.0 (SAS Institute Inc., Cary, NC, USA) and R version 2.10.1 [57]. LME models were fitted in R using the lmer-procedure in the LME4 library. Information about variance inflation due to collinearity (assessed from the general variance inflation factor, GVIF) was retrieved using the CAR library.

## Authors' contributions

TOH and LAV designed the study, all authors took part in field and laboratory work, but all measurements have been collected by GT. Analyses of the results were conducted by GT and TOH. All have taken part in the writing of the paper. All authors have read through and approved the last version.

## Supplementary Material

Additional file 1 **Parameter estimates (± se) for the factors included in linear mixes models (see equation 1 in main text)**. Click here for file

Additional file 2 **Summary table for deme-wise linear regressions of ln(length) on degree days in the three treatment temperatures**. In **Table A2 **the intercept, slope (with SE), adjusted r^2 ^and N are given. The results are also visualised in **Figure A2**. Click here for file

Additional file 3 **Summary table for deme-wise linear regressions of ln(yolk sac area) on degree days in the three treatment temperatures**. In **Table A3 **the intercept, slope (with SE), adjusted r^2 ^and N are given. The results are also visualised in **Figure A3**. Click here for file
