# Supplementary material for: Contemporary divergence in early life history in grayling (Thymallus thymallus)
Source: BMC Evol Biol. 2011 Dec 13;11:360. doi: 10.1186/1471-2148-11-360 (PMC3252335; doi:10.1186/1471-2148-11-360)
Supplement: Additional file 1 — Parameter estimates (± se) for the factors included in linear mixes models (see equation 1 in main text). [file 1471-2148-11-360-S1.PDF]

Additional file 1

Table A1: Parameter estimates (± se) for the factors included in the models fitted to the data (see equation 1 in main text). Linear mixed models (LME) were used for investigating variation in length and yolk sac area over time. Replicate, nested under deme, was used as a random factor in the analyses. In order to ease reconstruction of the yolk-sac area model, an ordinary quadric polynomial function was fitted for the ln(°d) term. This approach differs somewhat from the orthogonal parameterisation approach that is reported in Table 2. The Treatm*Deme*ln(°d)^2 interaction effect is not included for the ordinary polynomial approach as this full model did not converge.

°d = degree-days. Treatments are C = cold, M = medium, W = warm. Demes are VAL = Valåe, STEIN = Steinbekken, SAND = Sandbekken, and Hyrjon is the default deme.

Trait	Term	Estimate	SE
**ln(length)**	Intercept	-1.2867	0.3704
R^2 = 0.712, N = 709	Treatm(M)	0.5218	0.4987
	Treatm(W)	-1.7155	0.5906
	Deme(VAL)	0.3457	0.4044
	Deme(STEIN)	1.2837	0.4178
	Deme(SAND)	1.0200	0.4140
	°d	0.6883	0.0682
	Treatm(M)*Deme(VAL)	0.3256	0.5974
	Treatm(W)*Deme(VAL)	1.5474	0.8037
	Treatm(M)*Deme(STEIN)	-0.9343	0.7344
	Treatm(W)*Deme(STEIN)	0.2492	0.8303
	Treatm(M)*Deme(SAND)	-1.6484	0.7250
	Treatm(W)*Deme(SAND)	-0.2213	0.7655
	Treatm(M)*°d	-0.0779	0.0914
	Treatm(W)*°d	0.3411	0.1097
	Deme(VAL)*°d	-0.0577	0.0743
	Deme(STEIN)*°d	-0.2221	0.0766
	Deme(SAND)*°d	-0.1776	0.0759
	°d*Treatm(M)*Deme(VAL)	-0.0622	0.1093
	°d*Treatm(W)*Deme(VAL)	-0.2850	0.1494
	°d*Treatm(M)*Deme(STEIN)	0.1672	0.1352
	°d*Treatm(W)*Deme(STEIN)	-0.0409	0.1554
	°d*Treatm(M)*Deme(SAND)	0.2964	0.1335
	°d*Treatm(W)*Deme(SAND)	0.0488	0.1431

**ln(yolk-sac area)**	Intercept	-198.19	63.56
R^2 = 0.772, N = 707	Treatm(M)	-89.53	64.38
	Treatm(W)	20.89	79.76
	Deme(VAL)	-201.42	66.21
	Deme(STEIN)	-126.29	67.07
	Deme(SAND)	-41.95	65.72
	ln(°d)	75.54	23.49
	ln(°d)2	-7.13	2.17
	Treatm(M)*Deme(VAL)	-0.35	0.10
	Treatm(W)*Deme(VAL)	-0.50	0.12
	Treatm(M)*Deme(STEIN)	-0.16	0.11
	Treatm(W)*Deme(STEIN)	-0.45	0.14
	Treatm(M)*Deme(SAND)	-0.18	0.11
	Treatm(W)*Deme(SAND)	-0.55	0.14
	Treatm(M)*ln(°d)	35.19	23.68
	Treatm(W)*ln(°d)	-4.81	29.99
	Treatm(M)*ln(°d)^2	-3.45	2.18
	Treatm(W)*ln(°d)^2	0.16	2.82
	Deme(VAL)*ln(°d)	76.33	24.43
	Deme(STEIN)*ln(°d)	48.64	24.77
	Deme(SAND)*ln(°d)	17.09	24.26
	Deme(VAL)*ln(°d)^2	-7.23	2.25
	Deme(STEIN)*ln(°d)^2	-4.68	2.29
	Deme(SAND)*ln(°d)^2	-1.74	2.24
