# Supplementary material for: Contemporary divergence in early life history in grayling (Thymallus thymallus)
Source: BMC Evol Biol. 2011 Dec 13;11:360. doi: 10.1186/1471-2148-11-360 (PMC3252335; doi:10.1186/1471-2148-11-360)
Supplement: Additional file 2 — Summary table for deme-wise linear regressions of ln(length) on degree days in the three treatment temperatures. In Table A2 the intercept, slope (with SE), adjusted r2 and N are given. The results are also visualised in Figure A2. [file 1471-2148-11-360-S2.PDF]

Additional file 2

Table A2: Summary table for deme-wise linear regressions of ln length on degree days in the three treatment temperatures. Intercept, slope (with SE), adjusted r^2 and N are given. The results are also visualised in Figure A2 (below).

Treatment	Deme	Intercept	Slope	SE	r^2 adjusted	N
Warm	Hyrjon	-2.925	1.015	0.152	0.577	33
	Valåe	-1.166	0.697	0.065	0.780	33
	Steinbekken	-1.461	0.765	0.066	0.833	28
	Sandbekken	-2.184	0.897	0.083	0.773	35
Medium	Hyrjon	-0.755	0.608	0.044	0.775	56
	Valåe	-0.091	0.490	0.033	0.761	70
	Steinbekken	-0.401	0.553	0.062	0.657	42
	Sandbekken	-1.401	0.731	0.083	0.669	39
Cold	Hyrjon	-1.033	0.643	0.086	0.560	47
	Valåe	-0.641	0.577	0.027	0.798	122
	Steinbekken	-0.014	0.468	0.029	0.707	105
	Sandbekken	-0.314	0.520	0.026	0.789	107

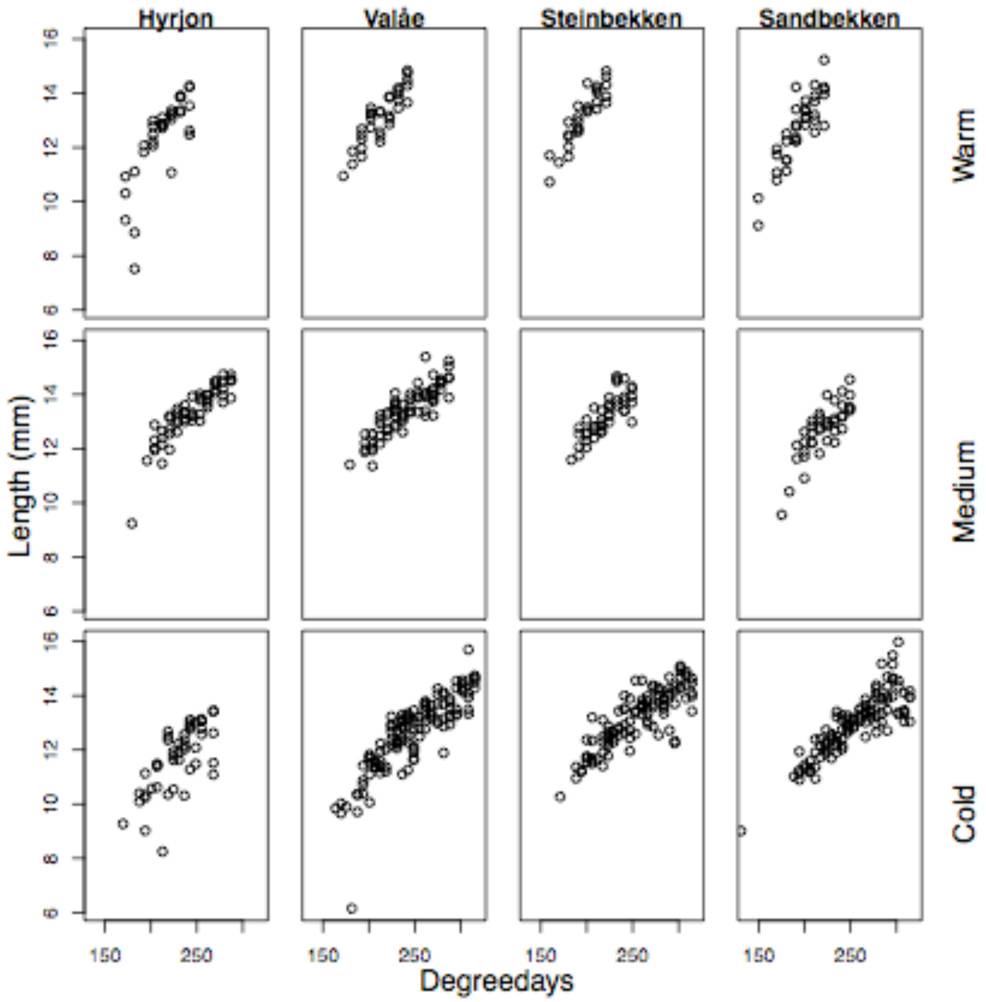

Figure A2: Scatterplots showing the length (mm) of each sampled individual from each deme at each treatment temperature (cold, medium and warm).
