# Supplementary material for: Contemporary divergence in early life history in grayling (Thymallus thymallus)
Source: BMC Evol Biol. 2011 Dec 13;11:360. doi: 10.1186/1471-2148-11-360 (PMC3252335; doi:10.1186/1471-2148-11-360)
Supplement: Additional file 3 — Summary table for deme-wise linear regressions of ln(yolk sac area) on degree days in the three treatment temperatures. In Table A3 the intercept, slope (with SE), adjusted r2 and N are given. The results are also visualised in Figure A3. [file 1471-2148-11-360-S3.PDF]

Additional file 3

Table A3: Summary table for deme wise linear regressions of ln(yolk sac area) (mm^2) on degree days in the three treatment temperatures. Intercept, slope (with SE), adjusted r^2 and N are given. The results are also visualised in Figure A3 (below).

**Treatment**	**Deme**	**Intercept**	**Slope**	**SE**	**r^2 adjusted**	**N**
Warm	Hyrjon	10.897	-1.787	0.41	0.360	33
	Valåe	21.012	-3.747	0.41	0.721	33
	Steinbekken	10.537	-1.770	0.31	0.533	28
	Sandbekken	11.186	-1.912	0.29	0.547	35
Medium	Hyrjon	18.363	-3.148	0.41	0.517	56
	Valåe	29.558	-5.287	0.34	0.776	70
	Steinbekken	19.596	-3.423	0.40	0.643	42
	Sandbekken	15.790	-2.731	0.41	0.529	39
Cold	Hyrjon	8.064	-1.230	0.28	0.312	47
	Valåe	19.872	-3.449	0.25	0.611	122
	Steinbekken	22.974	-4.015	0.22	0.763	105
	Sandbekken	17.784	-3.065	0.21	0.673	107

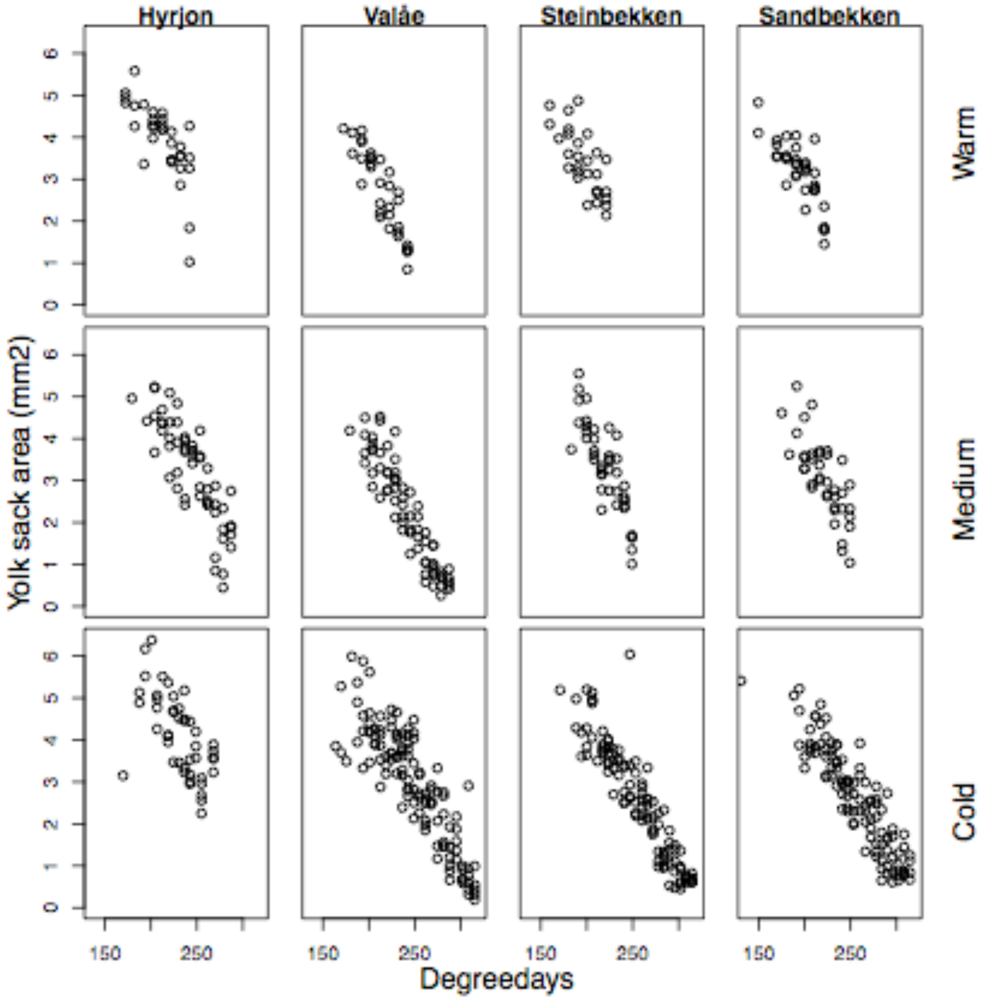

Figure A3: Scatterplots showing the yolk sac area (mm^2) of each sampled individual from each deme at each treatment temperature (cold, medium and warm).
